# Three-Dimensional Cell Drawing Technique in Hydrogel Using Micro Injection System

**DOI:** 10.3390/mi13111866

**Published:** 2022-10-30

**Authors:** Takuya Shinagawa, Shogo Miyata

**Affiliations:** 1 Graduate School of Science and Technology, Keio University, 3-14-1 Hiyoshi, Yokohama 223-8522, Japan; 2 Department of Mechanical Engineering, Faculty of Science and Technology, Keio University, 3-14-1 Hiyoshi, Yokohama 223-8522, Japan

**Keywords:** neuron, cell patterning, three-dimensional tissue, neuronal differentiation, hydrogel

## Abstract

Fabrication of three-dimensional tissues using living cells is a promised approach for drug screening experiment and in vitro disease modeling. To study a physiological neuronal function, three-dimensional cell patterning and construction of neuronal cell network were required. In this study, we proposed a three-dimensional cell drawing methodology in hydrogel to construct the three-dimensional neuronal cell network. PC-12 cells, which were used as neuronal cell differentiation model, were dispensed into a collagen hydrogel using a micro injector with a three-dimensional position control. To maintain the three-dimensional position of cells, atelocollagen was kept at sol-gel transition state during cell dispensing. As the results, PC-12 cells were patterned in the atelocollagen gel to form square pattern with different depth. In the patterned cellular lines, PC-12 cells elongated neurites and form a continuous cellular network in the atelocollagen gel. It was suggested that our three-dimensional cell drawing technology has potentials to reconstruct three-dimensional neuronal networks for an investigation of physiological neuronal functions.

## 1. Introduction

Neurons are composed of the nuclear periphery, which carries genetic information, and dendrites and axons, which get/deliver electrical signals from/to other neurons. Neurodegenerative diseases such as Alzheimer’s disease (AD), Parkinson’s disease (PD), Huntington’s disease (HD), and amyotrophic lateral sclerosis (ALS) are diseases that reduce the quality of life, to decrease daily physical activities, and have become major problems as society ages. The treatment of these neurodegenerative diseases relies on drugs, still has been difficult to recover sufficient physiological functions. Recently, for drug screening, drug discovery and in vitro diseases modeling, there exist approaches called Lab-On-a-Chip and Organs-On-Chip, in which cells are organized two or three–dimensionally to mimic physiological functions in vivo [1]. Studies on heart chips [2,3,4,5,6], liver chips [7,8], kidney chips [9,10], pancreas chips [11,12], blood–brain barrier (BBB) chips [13,14], have been already reported. There have also been approaches to mimic neural networks in vitro by controlling the position of neurons and the direction of neurite growth. Control of cell positioning and neurite outgrowth direction were enabled by cell culture substrates containing microstructures or micropatterns. These microstructures could be generated by soft lithography [15,16,17,18] and laser etching [19]. There also have been direct cell patterning approach using microinjection of cells [20]. Although these approaches are superior in mimicking neuron–neuron connections, they could not mimic and reconstruct in vivo three-dimensional neuron network because these approaches could only control two-dimensional structures.

Although it was reported that collagen hydrogels were suitable for three-dimensional culture of neuronal cells [21,22,23], three-dimensional cell patterning in hydrogels could not be performed. Three-dimensional printing based on an inkjet technology [24,25,26] enabled three–dimensional cell assembly. This approach was superior for accumulating cells three–dimensionally to fabricate cell aggregates, however, were difficult to place cells three-dimensionally inside scaffold materials for simulating a three-dimensional neural network. Furthermore, 3D printing of living cells has risks to damage cells by high speed and pressure dispensing from the inkjet nozzle.

In this study, we proposed a novel method for three-dimensional cell patterning in hydrogel to reconstruct a neuronal network. To avoid cell damage during cell patterning in hydrogels, micro injection tool was used for cell patterning. For fundamental study, PC-12 cells simulating neuronal differentiation were patterned three-dimensionally in collagen hydrogels. Verification of collagen gel types for cell patterning and evaluation of three-dimensional neuronal network construction were evaluated.

## 2. Materials and Methods

### 2.1. Theree-Dimensional Cell Drawing System

**Figure 1:**
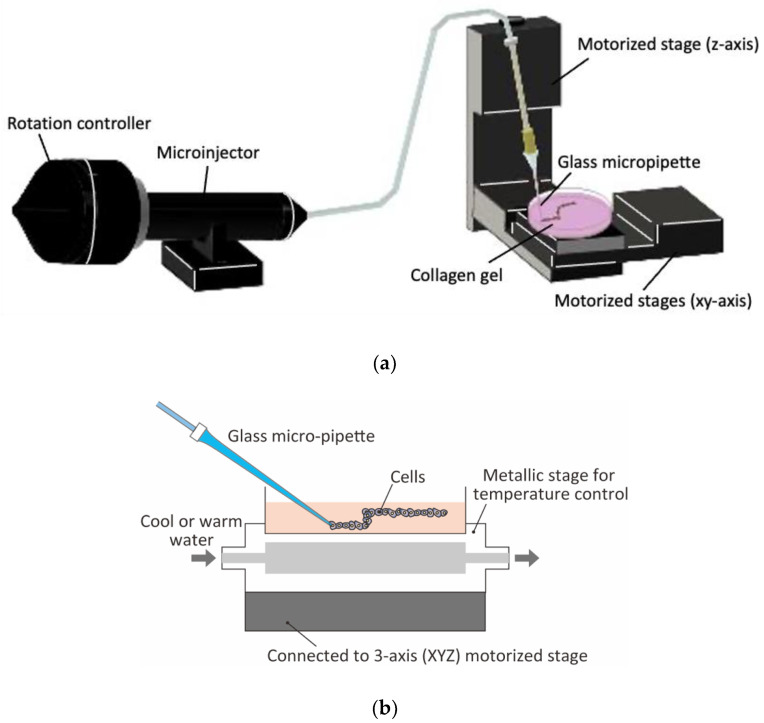
Micro-injection and three-dimensional position control system for three-dimensional cell drawing. (**a**) Experimental setup consisting of micro-injector and 3-axis motorized stages, (**b**) temperature control of system for collagen sol/gel.

To perform the three-dimensional patterning of cells in hydrogels, cell suspension was injected into hydrogel with a three-dimensional position control of injector nozzle to construct three-dimensional cell patterns. We named this novel cell patterning technique as the “cell drawing technique”. Three-dimensional cell drawing system was developed using a micro-injection system for cell suspension into hydrogel with three-dimensional position control (Figure 1a). The micro injection of cell suspension was performed by an oil-filled manual microinjector (CellTram 4 Oil, Eppendorf AG, Hamburg, Germany) with glass micro-pipette. The glass micro-pipette was made from a 1-mm diameter glass capillary (GD-1, Narishige, Tokyo, Japan) sharpened by a commercial puller (PC-10, Narishige, Tokyo, Japan). The diameter of micro-pipette tip was approximately 90 mm. To control the injection rate of cell suspension, the rotation of knob for microinjector was controlled by a microscope focus controller (MSS-FC, Chuo Precision Industrial, Tokyo, Japan) connected to a personal computer with a terminal software (Tera Term, open source). The cell suspension was injected via glass micro-pipette into neutral collagen sol or gel on the metallic stage whose temperature was controlled by circulating heating-cooling water (Figure 1b). The three-dimensional position of metal stage was controlled by 3-axis motor driven stage (Opto-Sigma, Tokyo, Japan) to “draw” the three-dimensional cell patterns. The motor driven stages were connected to a personal computer with a commercial control software (LabVIEW 2015, National Instruments, Austin, TX, USA).

### 2.2. Cell Culture

PC-12 cells (neuron-like cell line; Riken Bioresource Center, Tsukuba, Japan) was used to reconstruct neuronal-like network in a collagen gel. PC-12 cells were cultured in Dulbecco’s Modified Eagle Medium (DMEM) with 10% horse serum (HS) and 10% fetal bovine serum (FBS) to prepare sufficient cell number for the three-dimensional cell drawing experiments. For the cell drawing experiments, the cells were suspended in a fresh culture medium at a concentration of 1 × 10^6^ cells/mL. After injection into the collagen gel, the cells were cultured in DMEM + 10% HS + 10% FBS + 5 nM Nerve Growth Factor (mNGF 2.5S, Alomone Labs, Jerusalem, Israel) to induce neuronal differentiation.

### 2.3. Verification of Collagen Gel Types for Scaffold Material System

To determine an appropriate collagen gel type for the three-dimensional cell injection, native collagen and atelocollagen were tested for the hydrogel material. Type I acid-soluble collagen (IAC-30, Koken, Tokyo, Japan) and type I pepsin-solubilized collagen (IPC-30, Koken, Tokyo, Japan) in acidic solutions at 3.0 mg/mL were used as native collagen and atelocollagen, respectively. 2.4 mg/mL neutralized collagen solution was prepared from the 3.0 mg/mL acidic collagen solution and poured into a *f*100 petri dish to form a collage gel with 2.0 mm of thickness. The neutralized collagen solutions in petri dishes were gelled at 37 °C for 20 min on the metallic stage of cell-drawing system.

**Figure 2:**
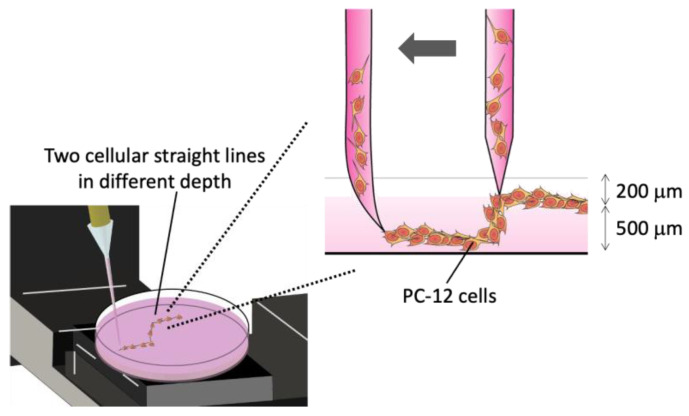
Schematic of micro-injection of PC-12 cells to draw two straight lines of cells with different depth in a collagen gel.

Following the preparation of collagen hydrogels in petri dishes, PC-12 cell suspension was dispensed in the gel to draw two straight lines of different depth (Figure 2). At first, the tip of glass micro-pipette was positioned at the depth of about 200 mm from the gel surface. Secondly, the tip was moved horizontally to draw a 10 mm straight line with dispensing cell suspension, descended for vertically for 500 mm, and moved horizontally to draw a 10 mm straight line with dispensing the cell suspension. The rate of tip movement was set at 500 mm/s and dispensing rate of cell suspension was set at 0.192 μL/s. After cell drawing, the cell-patterned collagen gels were cultured for 4 days in the neuronal differentiation medium. At the end point of culture, day 4, the difference in focal point position of each cellular straight pattern and geometry of cellular patterns were evaluated by a phase-contrast microscope (TE-2000, Nikon, Tokyo, Japan) attached with a focus controller (MSS-FC, Chuo Precision Industrial, Tokyo, Japan).

### 2.4. Verification of Gelation Conditions during Three-Dimensional Cell-Drawing

From the results in verification of collagen gel type, the atelocollagen gel was used for a scaffold material of three-dimensional cell drawing. In this section, the effect of gelation condition on the cell drawing was evaluated.

Firstly, 2.4 mg/mL neutralized solution of atelocollagen was prepared as described in Section 2.3. Before the three-dimensional cell drawing, the neutralized collagen solutions were incubated at 25, 30, 35 °C for 20 min, respectively. Following the incubation, the cell suspension was dispensed in the collagen sols or gels (depending on incubation temperatures) to draw a square pattern with four sides of different heights.

**Figure 3:**
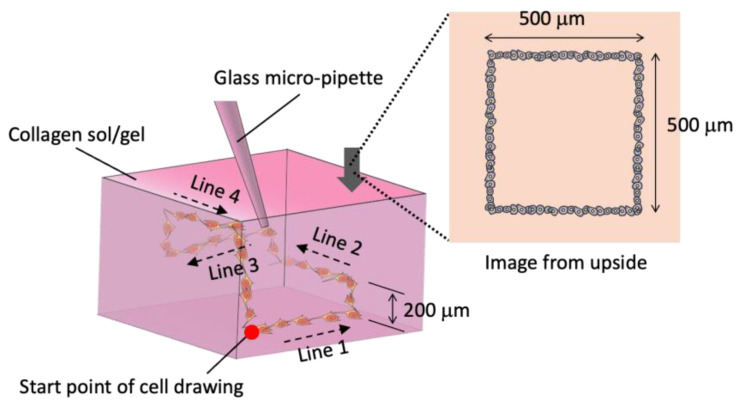
Square pattern drawing of PC-12 cells with four sides of different depth.

As shown in Figure 3, the tip of glass micro-pipette was positioned in the depth of 1 mm from the gel surface. Next, the tip was moved along with *x*-axis for 500 mm (Line 1), elevated along with *z*-axis for 200 mm, moved along with *y*-axis for 500 mm (Line 2), elevated for 200 mm, moved along with *x*-axis for −500 mm (Line 3), elevated for 200 mm, moved along with *y*-axis for −500 mm (Line 4), and descended for 600 mm to reach the start positions. During the drawing of the square pattern, the tip was moved at the rate of 500 mm/s and the cells suspensions was injected at the rate of 0.192 μL/s.

After the dispensing of cell suspensions in the collagen sol or gel, the cell-injected gels were incubated at 37 °C for 30 min for fully gelation, and cultured in a similar condition as described in Section 2.3. The geometry of cellular patterns was observed using a phase-contrast microscope (TE-2000, Nikon, Tokyo, Japan).

### 2.5. Theree-Dimensional Cell Drawing of Neuronal Cells and Differentiation of PC-12 Cells

From the results of validation experiments for collagen gel types and gelation conditions, the PC-12 cells were dispensed into the atelocollagen gel at 30 °C for 20 min. The neuronal differentiation and neuronal network formation of PC-12 cells in the three-dimensional pattern were evaluated.

To form the three-dimensional patten in the collagen gel, the tip of glass micro-pipette was moved to draw the same square pattern described in Section 2.4. The movement rate of tip was set at 500 mm/s and the injection rate of cell suspension were set at 0.096, 0/196, 0.48 mL/s to change cell concentrations in the drawn lines. After cell drawing, the cell-patterned collagen gels were incubated at 37 °C for 30 min and cultured for 4 days in the neuronal differentiation medium. The cellular patterns were evaluated using a phase-contrast microscope (TE-2000, Nikon, Tokyo, Japan) and image-processing software (ImageJ, National Institute of health, Bethesda, MD, USA).

**Figure 4:**
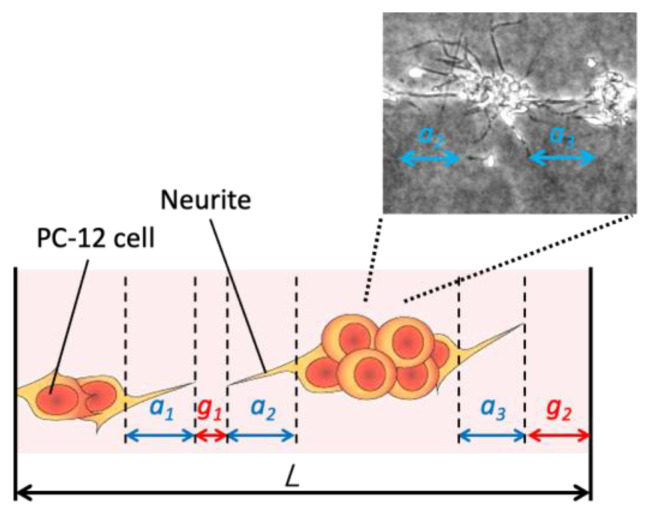
Evaluation of neurite formation of PC-12 cells and continuity of cellular pattern in a collagen gel. Neurite length was defined as the length of neurites connecting to adjacent cell aggregates.

For the fundamental validation of our proposed method, the construction of neuronal cell network was evaluated from phase-contrast microscopic images. Neuronal differentiation and network formation were evaluated by image-based analysis. Neuronal differentiation of PC-12 cells could be evaluated by the neurite generation [27,28]. In this study, the length of neurite, *a*, was defined and measured as the length of neurite connecting the adjacent cell aggregates (Figure 4). The gap length of cellular patterns, *g*, was also measured. Both measurements were performed in each side of square pattern. Rate of total neurite length and continuity of cellular pattern, *N* and *C*, were calculated as follows;

(1) $$N=\sum a_{n}/L,\;C=1-\sum g_{n}/L$$

where *L* is the length of side of square patterns.

### 2.6. Statistical Analysis

Most of the data are representative of two to three individual experiments with similar results. For each experimental group, five to seven samples (*n* = 5 or 10) were analyzed, and each data point represents the mean and standard deviation.

## 3. Results

### 3.1. Effect of Collagen Gel Types on Three-Dimensional Cell Drawing

To validate appropriate collagen gel types, PC-12 cells were injected into two types of collagen gel to form straight lines with different depth. First cellular line (bottom line) was drawn at the depth of 1 mm from gel surface and second line (upper line) was at the depth of 0.5 mm from the gel surface in both of collagen gel types; native collagen and atelocollagen.

**Figure 5:**
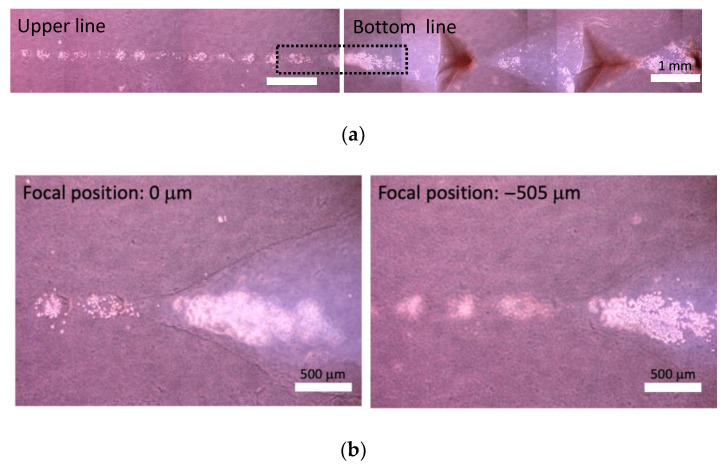
Phase-contrast images of straight lines at different depth in native collagen gel. (**a**) Gross images of upper and bottom lines, (**b**) enlarged images at different focal positions indicated by dashed line in the gross image.

**Figure 6:**
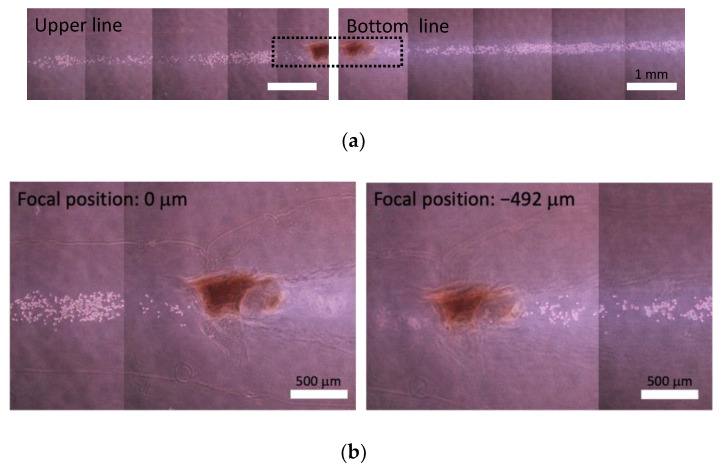
Phase-contrast images of straight lines at different depth in atelocollagen gel. (**a**) Gross images of upper and bottom lines, (**b**) enlarged images at different focal positions indicated by dashed line in the gross image.

**Figure 7:**
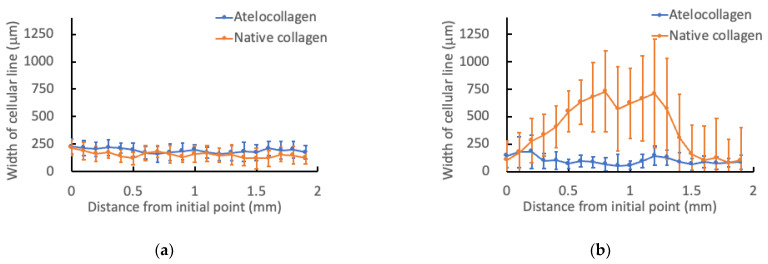
Width of cellular lines in collagen gels at (**a**) upper and (**b**) bottom lines. Mean ± S.D., *n* = 10.

The differences in focal position of these two lines was 483 ± 24 mm (*n* = 10) and 491 ± 24 mm (*n* = 9) in native collagen and atelocollagen gels, respectively (Figure 5 and Figure 6). From macroscopic view, straight lines were formed both in native collagen and atelocollagen gels. However, at the microscale, the straight lines in native collagen gel were intermittently drawn, and the cracks made by glass micropipette movement was observed (Figure 5). On the other hand, the straight lines could be drawn both in upper and bottom layers in atelocollagen gel (Figure 6). Furthermore, the width of cellular lines was different between upper and bottom lines in the native collagen gel whereas the significant differences in width were not observed in the atelocollagen gel (Figure 7).

### 3.2. Relationships between Gelation Condition and Maintenance of Cellular Pattern

**Figure 8:**
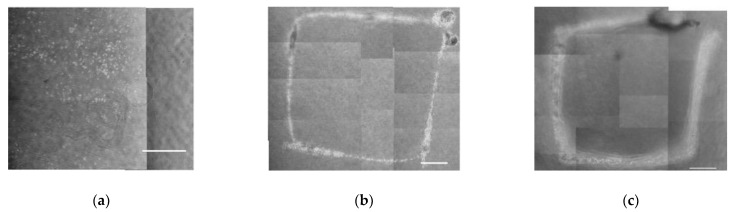
Phase-contrast images of square pattern of PC-12 cells injected in atelocollagen sol or gel at (**a**) 25 °C, (**b**) 30 °C, and (**c**) 35 °C. Scale bar = 1 mm.

To determine an appropriate gelation condition of collagen gel, the three-dimensional cell drawing experiments were performed under different gelation temperature. At lower temperature, 25 °C, the square pattern of cells was not maintained and cells were dispersed during the cell injection in collagen gel (Figure 8a). The square patterns could be formed and maintained in the collagen gels gelled at over 30 °C (Figure 8b,c). The square pattern of cells could be formed without crack or scratch by the tip of glass micropipette at 30 °C, whereas the cracks and scratches were observed in the pattern at 35 °C.

### 3.3. Neuronal Differentiation and Network Construction of PC-12 Cells in Three Dimensional Pattern

Finally, PC-12 cells were injected into an atelocollagen gel treated at 30 °C to form a square pattern, and were cultured to induce neuronal differentiation. To evaluate cell concentrations on neuronal differentiation and network formation, three injection rate of cell suspensions were evaluated.

**Figure 9:**
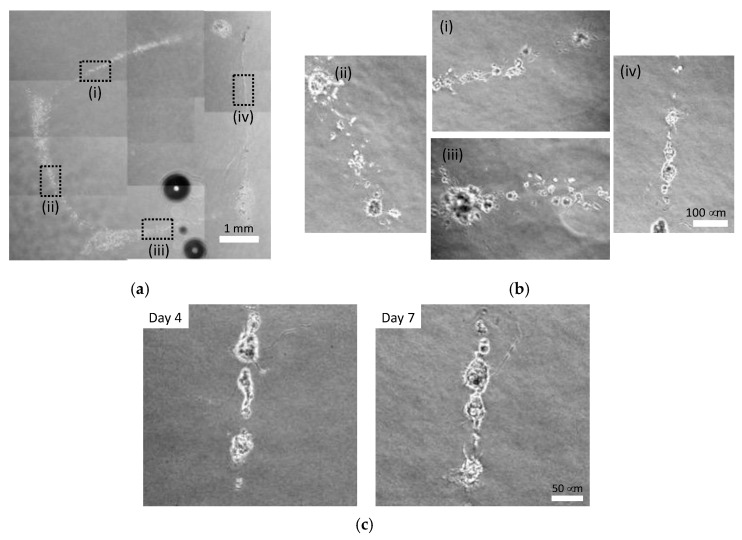
Phase-contrast images of square pattern of PC-12 cells injected at 0.096 mL/s in atelocollagen gel. (**a**) Gross image, (**b**) enlarged images of (i) Line 1, (ii) Line 2, (iii) Line 3 and (iv) Line4 of square pattern on day 7; (**c**) Neurite growth during the culture time.

**Figure 10:**
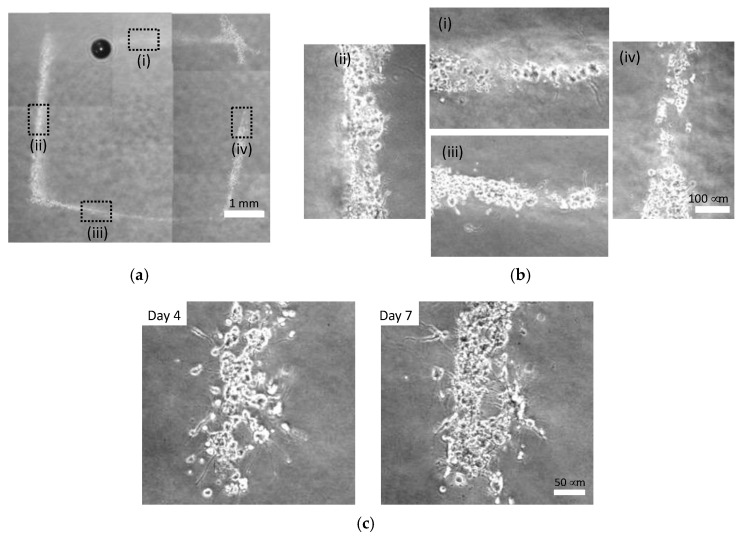
Phase-contrast images of square pattern of PC-12 cells injected at 0.196 mL/s in atelocollagen gel. (**a**) Gross image, (**b**) enlarged images of (i) Line 1, (ii) Line 2, (iii) Line 3 and (iv) Line4 of square pattern on day 7; (**c**) Neurite growth during the culture time.

**Figure 11:**
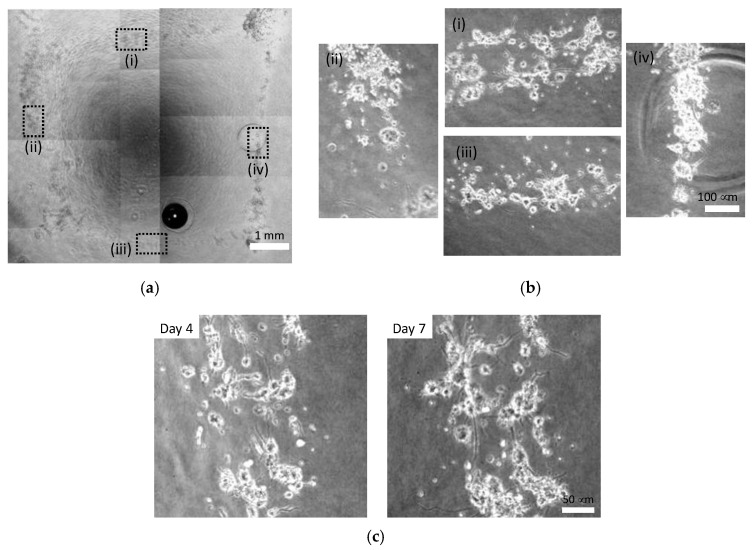
Phase-contrast images of square pattern of PC-12 cells injected at 0.480 mL/s in atelocollagen gel. (**a**) Gross image, (**b**) enlarged images of (i) Line 1, (ii) Line 2, (iii) Line 3 and (iv) Line4 of square pattern on day 7; (**c**) Neurite growth during the culture time.

Cellular patterns could be formed at the injection late of 0.096 and 0.196 mL/s, whereas cells were dispersed and width of cellular lines were larger at 0.480 mL/s (Figure 9, Figure 10 and Figure 11). The cells injected at 0.096 mL/s formed small aggregates and thinner lines compared to those injected at other rates (Figure 9). The cells injected at 0.196 mL/s tended to form continuous lines with small cell aggregates (Figure 10). The cells injected at 0.480 mL/s dispersed to form thicker lines and formed small aggregates in each line (Figure 11). PC-12 cells in atelocollagen gel in all experimental conditions extended neurites with an increase in the culture time for all experimental conditions.

**Figure 12:**
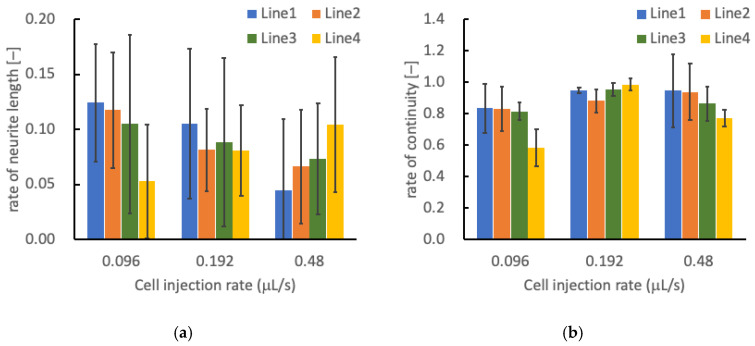
Relationships between cell injection rate and (**a**) neurite growth ratio, and (**b**) continuity ratio in square patterns of PC-12 cells. Mean ± S.D., *n* = 5.

Rate of neurite length in each cellular line tended to increase with a decrease in the depth of line within square pattern made at injection rate of 0.096 mL/s, whereas tended to decrease in the pattern at the injection rate of 0.480 mL/s (Figure 12a). The rate of neurite length in each line of square pattern was similar when the cellular pattern was made at the injection rate of 0.196 mL/s. The rate of continuity in each cellular line tended to increase with a decrease in the depth of line within square pattern made at injection rate of 0.096 and 0.480 mL/s (Figure 12b). The rate of continuity in each line of square pattern was also similar when the cellular pattern was made at the injection rate of 0.196 mL/s.

## 4. Discussion

Tissue engineering approaches have enabled three-dimensional cell culture to replicate biological tissues in a human body. Various organs and tissues were replicated by culturing cells on porous polymer materials or hydrogels as scaffolds. Organoid culture is also one of the promising approaches to reconstruct micro biological tissues for drug screening platform [1]. Organoid culture of neuronal cells has been performed as various forms: sliced tissue culture, microfluidic culture, vascularized culture, etc [29]. These approaches were superior for mimicking native neuronal structure in brain to study neuronal function and diseases development. However, precise monitoring of the action potential propagation was difficult in these neuronal organoid cultures because organoid cultures were formed based on cell aggregates. To solve this problem, neuronal cell patterning methods have been reported to observe the propagation of signal and action potential in neuronal networks [18]. In the previous studies, neuronal cells were patterned in a two-dimensional form, therefore three-dimensional cell patterning methodology to replicate physiological neuronal cell condition was required. Our method proposed in this study showed a potential to breakthrough this problem.

Firstly, we validated the appropriate scaffold material for three-dimensional cell patterning. Collagen hydrogels have been used wifely for three-dimensional culture [30,31]. Acid-soluble collagen and atelocollagen were used to form hydrogels in this study. From the results of this study, soft hydrogel, atelocollagen was appropriate for cell drawing with injection into hydrogel without scratch marks (Figure 6). Furthermore, sol-gel transition condition of atelocollagen could keep three-dimensional positions of cells injected by glass micropipette without scratch marks using a precise temperature control for collagen gelation (Figure 8). It was suggested that the position of cells was retained in collagen gel without disturbing the cell pattern at near the sol–gel transition temperature of agarose gel.

The effect of cell concentration on the neuronal differentiation and construction of cellular network in patterned lines were evaluated. PC-12 cells elongated their neurite in the atelocollagen gel to form the cell networks in three-dimensional conditions. Under low cell concentration, PC-12 cells formed small aggregates and elongated their neurites at random direction without connecting with adjacent cells. Therefore, the rate of neurite grow length along with patterned lines tended to show lower values. The cell-patterned lines were intermittent because the injected cells formed small aggregates. The cells patterned with higher concentration also showed lower value for the rate of neurite growth length, however this might be due to different factors. The cells injected with higher flow rate dispersed widely in the hydrogel and the patterned lines of cell population were disturbed to form large cell aggregates. Therefore, the rate of neurite growth tended to show lower values as compared to other injection rates. In the cell-patterned lines at medium cell concentration, the neurite growth ratio tended to show higher values and did not change depending on the difference in the depth of lines (Figure 12). The rate of continuity also did not change depending on the line depth.

In this study, we used PC-12 cell as neuronal differentiation culture model to validate our three-dimensional cell drawing technology. PC-12 cells have been used widely for investigations about neuronal differentiation [27,28,32]. However, the PC-12 cells are derived from rat pheochromocytoma with poor potentials to mimic electrical activities as neurons. Therefore, to validate our proposed method for a three-dimensional neuronal network construction, validation experiments using native neuronal cells with monitoring a transition of active potential would be required. In addition, the possibilities of drawing complex cellular pattern and connections of neuronal networks should be evaluated by immunofluorescence staining. Although the requirement for validation experiments using neuronal cells, our method enabled firstly to reconstruct three-dimensional neuronal cell network in a hydrogel. Furthermore, our three-dimensional cell drawing method has a potential to construct a complex neuronal network with neuroglia and schwann cells using multi injection nozzles for cell drawing.

## 5. Conclusions

In this study, we proposed a three-dimensional cell drawing method for the construction of three-dimensional neuronal cell network. To pattern cells three-dimensionally in a hydrogel, cell suspension was injected into atelocollagen under sol-gel transition state using microinjector. The three-dimensional position of microinjector was controlled by 3-axis motorized-stages. Based on the results of this study, the PC-12 cells could be patterned three dimensionally in the atelocollagen gel. Furthermore, it was suggested that the PC-12 cell could be differentiated to elongate neurites and construct neuronal cell networks. Our cell drawing technique has potentials to construct the three-dimensional neuronal cell network simulating in vivo neuron functions.
